# Silicon Nanowire-Based Schottky Diodes for Enhanced Temperature Sensing and Extended Operable Range

**DOI:** 10.3390/s26030780

**Published:** 2026-01-23

**Authors:** Gheorghe Pristavu, Razvan Pascu, Melania Popescu, Monica Simion, Cosmin Romanitan, Iuliana Mihalache, Florin Draghici, Gheorghe Brezeanu

**Affiliations:** 1 Faculty of Electronics, Telecommunications and Information Technology, National University of Science and Technology Politehnica Bucharest, 060042 Bucharest, Romania; gheorghe.pristavu@upb.ro (G.P.); florin.draghici@upb.ro (F.D.); gheorghe.brezeanu@upb.ro (G.B.); 2 National Institute for Research and Development in Microtechnologies—IMT Bucharest, 077190 Bucharest, Romania; melania.popescu@imt.ro (M.P.); monica.simion@imt.ro (M.S.); cosmin.romanitan@imt.ro (C.R.); iuliana.mihalache@imt.ro (I.M.)

**Keywords:** Schottky diode, temperature sensor, silicon nanowires, contact inhomogeneity

## Abstract

This paper analyzes microstructural layout and electrical behavior of silicon nanowire-based Schottky diodes, for use as wide-domain temperature sensors. The employed nanostructured three-dimensional substrates provide larger contact areas and enable higher Schottky barrier heights, ultimately leading to a better operable temperature range. Two metal deposition techniques (Radio Frequency sputtering and Electron-beam evaporation) are used to fabricate experimental Schottky diode samples. Scanning electron microscopy, X-ray diffraction, and diffuse reflectance investigations are carried out in order to determine nanowire distribution and the influence of subsequent metal deposition. The analyses evince the formation of a slightly inhomogeneous contact. The findings are validated by a thorough electrical characterization over a wide temperature domain. Inhomogeneity models are used in order to determine the main device parameters and the bias regions where they can be used as precise temperature sensors. The sputtered sample exhibits the best sensitivity, between 1 and 1.4 mV/K, while excellent linearity (R^2^ > 99.5%) is obtained for Electron-beam evaporated devices. Both types of silicon nanowire-based Schottky diode sensors have 100–500K operable ranges, much larger than planar counterparts.

## 1. Introduction

Schottky diodes represent the essential part of many electronic and sensing applications due to their low forward voltage drop, quick switching speed, and very simple fabrication process [1,2]. Bulk Si Schottky diodes have been extensively characterized and integrated into mainstream electronics, such as temperature sensor devices [3,4], Radio Frequency (RF) circuits [5,6], and power electronics [7,8]. In the last decade, alternative approaches have been intensively studied in order to develop more efficient devices for applications in harsh environments. One of the outstanding solutions was to introduce wide-band gap semiconductors as a starting material. Accordingly, silicon carbide (SiC) based devices were fabricated and optimized for different applications, such as temperature sensors [9,10], gas sensors [11,12], or UV detectors [13,14]. SiC-based devices are particularly designed to operate at high temperatures and in harsh environments, but they entail a higher cost in terms of fabrication when we compare them with silicon-based ones.

The rapid progress of nanotechnology has led to the fabrication of nanostructured surfaces on bulk semiconductors, which are able to offer a number of advantages in terms of sensitivity, scalability, and compatibility with modern miniaturized systems. While planar semiconductor-based devices are most often preferred as reliable and advanced components in traditional electronics, silicon nanowires (SiNW) [15] or porous surfaces [16] could represent a significant advancement in nanoscale device technology. A recent study demonstrated the potential of SiNW in temperature sensing, leading to sensitivities of −1.8%/°C and rapid detection (response ≈ 0.2 s, recovery ≈ 1 s) in biological-scale mapping of heat sources [17]. Since temperature is the leading physiological indicator, SiNW technology can play a major role in the early detection and prevention of debilitating afflictions, especially for situations where continuous monitoring is recommended, such as for diabetic foot ulcerations [18].

With regard to Schottky diodes, the transition from planar to nanoscale-based devices is anticipated to be accelerated with the development of nanofabrication techniques, particularly in applications where accuracy, speed, and integration are crucial. They have garnered significant attention owing to their rectifying behavior, nanoscale integration capability, and enhanced Surface-to-Volume Ratio (SVR), which make them highly sensitive to environmental changes [19]. Their superior thermal conductivity and high SVR enable effective heat dissipation and improve device thermal stability [20]. This is essential for preserving performance over a wide range of temperatures, where traditional bulk devices could malfunction [21]. Accordingly, the devices fabricated on such substrates, particularly on SiNW, could represent a cost-effective solution for moderate to high-temperature applications. The SVR is known to have a direct impact on Schottky barrier height and saturation current, resulting in temperature sensitivity modifications [22]. Thus, the quality of the metal–semiconductor interface becomes crucial for such devices. The influence of surface states and interface inhomogeneity leads to the appearance of different carrier transport mechanisms, such as thermionic-field emission or tunneling, especially at lower diameters, showing deviations from pure thermionic emission [23]. While the Schottky diodes’ forward voltage temperature-dependent nature allows for precise sensing across a broad temperature range, their small size also enables fast thermal response [24]. However, smaller effective Schottky contacts typically reduce temperature sensitivity when the diode is biased at a constant current [25]. This drawback can be mitigated by properly engineering the metal/SiNWs Schottky contact, enabling the customization of sensitivity via barrier height tuning.

Recently, we compared the electrical properties of Schottky diodes fabricated with SiNWs in terms of saturation current and series resistance when a Radio Frequency (RF) sputtering metal deposition is used versus Electron-beam (E-beam) evaporation [26]. In this paper, we evaluate the wide-range temperature sensing performances of such SiNWs-based Schottky diodes in comparison to planar counterparts. The resulting devices demonstrated good sensing behavior over a wide-temperature range (100–500K).

## 2. Materials and Methods

### 2.1. SiNWs Fabrication via Metal-Assisted Chemical Etching (MACE)

**Figure 1:**
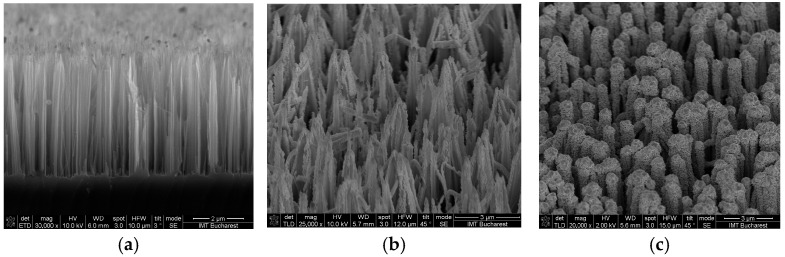
(**a**) SEM images of the as-prepared ~5 μm NWs, (**b**) after metal deposition using E-beam evaporation, and (**c**) after metal deposition using RF sputtering.

The Metal-Assisted Chemical Etching (MACE) process [15] is a simplified method for fabricating SiNWs that eliminates the need for hydrogen peroxide as an oxidizing agent. It integrates metal catalyst deposition with silicon etching in a single step using hydrofluoric acid (HF) and metal salts, primarily silver nitrate (AgNO_3_ purchased from Sigma-Aldrich, Taufkirchen, Germany). It starts with immersing the silicon substrate in an aqueous solution of AgNO_3_ and HF. Upon immersion, silver ions (Ag^+^) from AgNO_3_ undergo reduction at the silicon surface, forming silver nanoparticles (Ag NPs) that randomly distribute across the silicon substrate. This electroless deposition occurs through a galvanic displacement reaction, where silicon atoms donate electrons that reduce Ag^+^ ions to metallic silver. Silver nanoparticles (Ag NPs) form localized galvanic cells on the silicon surface, injecting holes into the silicon valence band due to their higher electronegativity. The radical NO_3_^−^ from AgNO_3_ aids this oxidation by accepting electrons from the silicon substrate. The injected holes oxidize silicon atoms beneath the silver nanoparticles, forming silicon dioxide (SiO_2_) or soluble silicon. Hydrofluoric acid dissolves the oxidized silicon, creating vertical etching channels as the nanoparticles sink into the substrate. One-step MACE produces extensive silver dendrite networks on silicon nanowires. These complex branched structures result from the continuous reduction and precipitation of silver ions during the etching process. Following etching, the excess silver dendrites must be removed by chemical cleaning, typically with concentrated nitric acid (HNO_3_) solutions. This cleaning step dissolves the bulk silver deposits while preserving silicon nanowire structures. Accordingly, using the above-described process flow, we obtained ~5 µm length SiNWs in 30 min of etching in a solution based on 0.06 M AgNO_3_ dissolved in 4.5 M HF (Figure 1a). Onto the resulting SiNWs, 600 nm of Ti was deposited using two distinct techniques: E-beam evaporation (Figure 1b) and RF sputtering (Figure 1c) to establish the best performing method of obtaining Schottky contacts on modified substrates.

A high-resolution Nova NanoSEM 630 microscope (FEI Company, Hillsboro, OR, USA) was used in order to evaluate the morphology and dimensions of the fabricated SiNWs. Figure 1 presents tilted-view SEM images of the fabricated SiNWs.

As shown in Figure 1b,c, the SiNWs bundles are more prominent due to the metal deposition, exhibiting a more compact surface, especially where an RF sputtering deposition technique was used (Figure 1c). Metal coatings obtained by the E-beam method (Figure 1b) looks like aggregated metal grains and are not consistent along the nanowire length. Instead, the RF sputtering technique ensures a more conformal metal deposition, covering almost the entire surface of SiNWs, resulting in greater active surface area. The RF sputtering layer also seems to be more tightly packed and thicker around the wires.

### 2.2. Si/SiNWs Schottky Diodes Fabrication

Both planar and SiNW-based Schottky diodes were fabricated for temperature sensing applications. The bulk silicon contains a highly doped substrate (~10^19^ cm^−3^) over which an epitaxial layer with a thickness of 10 µm and doping concentration around 5 × 10^14^ cm^−3^ was grown. Firstly, SiNWs were fabricated on the epi layer using the above-described MACE process, protecting the backside of the Si substrate with photoresist.

**Figure 2:**
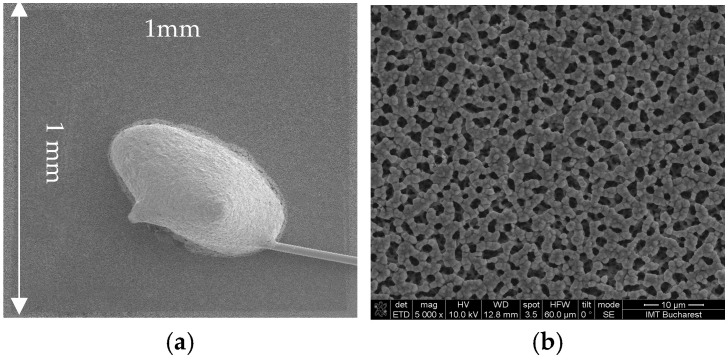
(**a**) Wire bonding process for packaged RF sputtering sample; (**b**) top-view of the surface Schottky contact.

After the SiNWS fabrication, the ohmic contact was achieved by using a successive metal deposition consisting of Ti (30 nm)/Au (300 nm) on the wafer backside. As described in [26], Schottky contacts (1 mm^2^, rectangular area) were fabricated using 600 nm of Ti by two deposition techniques: E-beam evaporation and RF sputtering. The fabricated devices were diced into chips and encapsulated in TO39 packages using wire-bonding technology. A top view of the most promising (RF sputtering) packaged sample is shown in Figure 2.

## 3. Results

### 3.1. Microstructural Investigations of Fabricated SiNW

#### 3.1.1. X-Ray Diffraction Analysis

**Figure 3:**
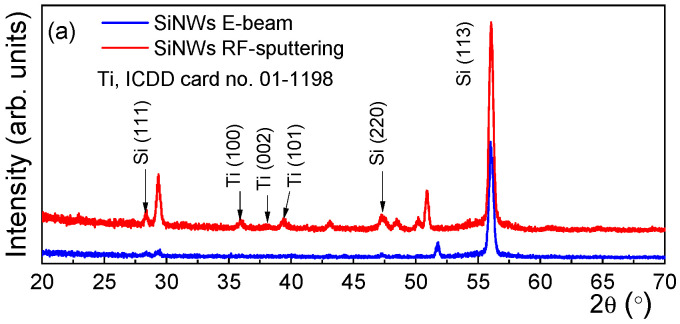

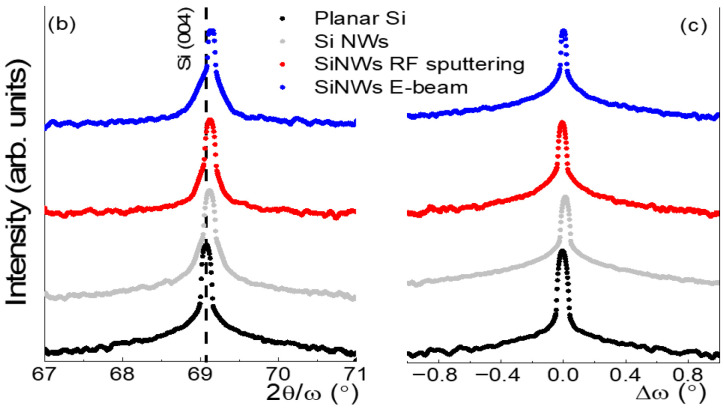
(**a**) The GI-XRD spectra for Ti/SiNWs Schottky contacts. High-resolution (**b**) 2θ/ω and (**c**) ω-scans.

XRD measurements were performed using a 9 kW Rigaku SmartLab diffractometer (Rigaku corp., Osaka, Japan) that uses a Cu Kα1 source that provides a monochromatic beam (λ = 0.15406 nm). The grazing incidence measurements were recorded by keeping the X-ray source at 0.5°. During the high-resolution measurement, a two-bounce Ge (440) monochromator was used at the incidence, while the detector remained open. The 2θ/ω and ω-scans were recorded around the Si (004) reflection, using a step of 0.004°. The SEM images indicated that the deposition with RF sputtering led to a Ti layer that seems to be more tightly packed and thicker around the wires. A more conformal deposition by RF-sputtering than E-beam is also indicated by grazing-incidence XRD patterns presented in Figure 3a, which show a crystallization of Ti deposited by RF-sputtering.

The diffraction peaks from Figure 3a, located at 2θ = 36.6, 38.2, and 39.5°, were assigned as (100), (002), and (101) reflections of hexagonal Ti (a = b = 0.292 nm, c = 0.467 nm), according to card no. 01-1198 of the ICDD (International Centre for Diffraction Data) database. On the other hand, the E-beam deposition did not generate Ti diffraction peaks. The XRD patterns recorded along 2θ/ω, shown in Figure 3b, reveal that the Ti deposition by either RF-sputtering or E-beam evaporation does not further affect the lattice constant of SiNWs. Moreover, the preservation of high SiNWs crystallinity after the deposition of Ti is noted. Additional information related to the NWs misorientation was assessed by rocking curves XRD spectra (Figure 3c) recorded around Si (004). It is well known that the ω-scans represent a measure of a NW misorientation in the out-of-the-plane [27,28]. The interpretation of the Full Width at Half Maximum (FWHM) showed that the Ti deposition did not induce an additional bending of the nanowires. The results suggest that a high verticality of the NWs is maintained even after Ti deposition.

#### 3.1.2. Diffuse Reflectance Analysis

Diffuse reflectance (R) was measured at room temperature using a Cary 5000 UV/Vis/NIR spectrophotometer with an integrating sphere (Agilent Technology, Santa Clara, CA, USA). The spectra were recorded in the 200 to 1500 nm range at 1 nm intervals. All spectra were baseline-corrected using 100% R recorded for the white polytetrafluoroethylene standard sample.

**Figure 4:**
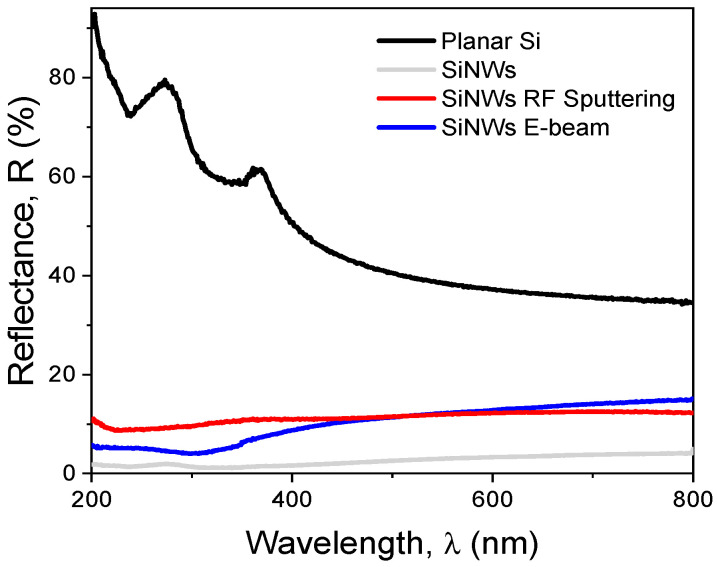
Reflectance of the surface of Planar Si and SiNW without (SiNWs) and with metal (SiNWs RF sputtering & SiNWs E-beam).

Figure 4 shows the total diffuse reflectance recorded on the surface of planar Si and on the SiNWs substrates used for the fabrication of Schottky diodes.

A strong suppression of the Si surface reflectance was observed across the entire UV and visible spectrum after the etching of the epi layer, confirming nanowire formation. Unlike planar Si, SiNWs are strongly absorptive of light with less than 5% reflection over the entire spectrum [29]. SiNWs have a textured geometry and graded refractive index, which facilitates trapping, scattering, and multiple internal reflections of light.

After the metal deposition on the SiNWs, the total reflectance of the surface is lower than 15% and approximately constant over broad visible and NIR wavelengths. In this case, the reflectance could serve as an indicator of the quality and homogeneity of the film deposition. Comparing the two deposition techniques, the RF sputtered substrate provides more uniform coverage of nanowire arrays due to higher and consistent reflectance values both at short (>10% for λ = 320 nm) and long wavelengths (<12% for λ > 400 nm). These results are consistent with the SEM morphological evaluation.

### 3.2. Modeling and Sensing Performances

#### 3.2.1. Temperature Dependent Electrical Characteristics and Conventional Methods for Contact Inhomogeneity Evaluation

**Figure 5:**
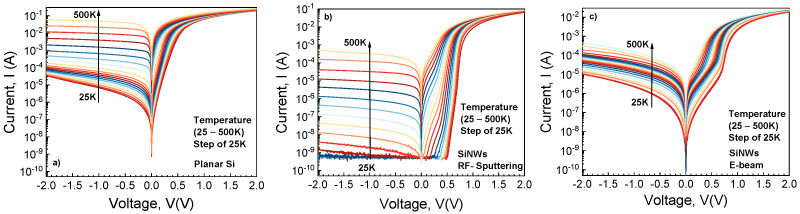
Experimental forward and reverse bias I-V characteristics of the SBDs at various temperatures: (**a**) Planar Si Schottky diode; (**b**) SiNWs Schottky diodes using RF sputtering as metal deposition, and (**c**) SiNWs Schottky diodes using E-beam as metal deposition.

Packaged samples from each batch were measured over 25–500K using a Keithley 4200 Semiconductor Characterization System (Keithley Instruments, LLC, Solon, OH, USA) coupled with a Janis closed-cycle refrigerator, CCS-450 (Janis Research Company LLC (now part of Lake Shore Cryotronics), Westerville, MA, USA). Current-Voltage (I–V) characteristics were acquired, in the −2V: 2V domain, with representative curves depicted in Figure 5.

Noticeably different electrical behaviors can be observed for each sample. The Planar Si diodes exhibit exponential forward I–V dependence only up to approx. 350K and very high reverse current, strongly increasing with temperature. Conversely, the SiNWs RF-Sputtering sample curves display a clear exponential portion even at 500K, with significantly lower reverse current across the entire temperature range. Curiously, the E-beam deposited nanowire diodes blend the behaviors of the previously presented samples. The visible impact of contact inhomogeneity depicts the transition from a low-barrier contact (akin to the planar SBDs) towards one with a higher Schottky Barrier Height (SBH) (like the SiNWs RF-Sputtering sample). The reverse current also displays this effect, resulting in a more tightly packed distribution. Its low and high temperature values correspond to those of planar and SiNWs RF-Sputtering, respectively.

**Figure 6:**
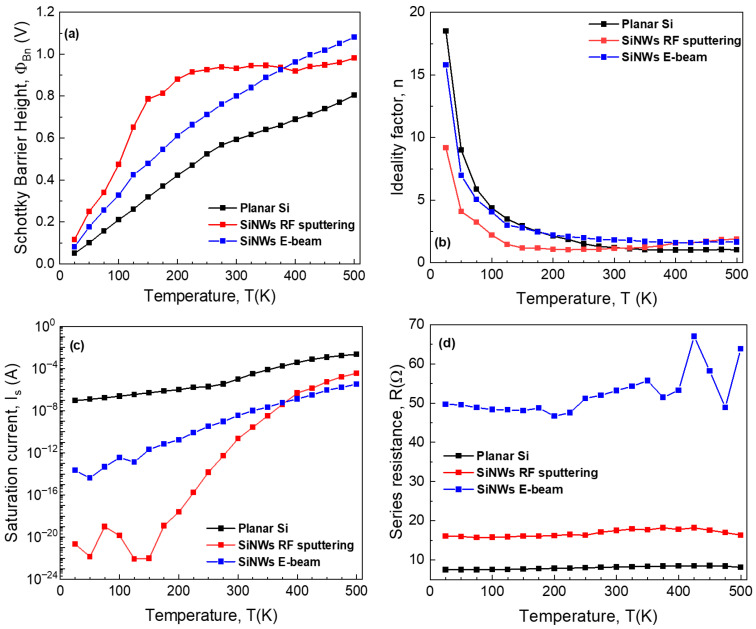
Temperature dependence of electrical parameters for fabricated SBDs: (**a**) SBH, (**b**) ideality factor, (**c**) saturation current, and (**d**) series resistance.

The main electrical parameters (SBH, ideality factor, saturation current, and series resistance) were extracted, and their temperature dependence is depicted in Figure 6. The Φ_Bn_ and *n* variations indicate a significant degree of contact inhomogeneity. As expected, the SiNWs samples achieve a higher overall SBH value, at the cost of higher series resistance.

In order to assess the practical performances of the samples, a thorough *p-diode* modeling [30] was performed over the entire 25–500K range, with results summarized in Table 1. Here, *m* represents the number of parallel-diodes. They model current flow through the overall inhomogeneous contact, acting as quasi-ideal Schottky diodes, each with their specific SBH, area (given by their non-uniformity parameter, *p_eff_*), and series resistance. In the case of SiNW-based devices, a parallel diode represents an aggregated zone, whose bundle of nanowires contributes unitarily to current conduction. Table 1 also gives the Φ_bn_ and *p_eff_* intervals for each sample.

The analysis confirmed that, for all samples, the low temperature/bias portions of forward characteristics are severely impacted by the inhomogeneous nature of the Schottky contact. As temperature and bias increase, the behavior of the samples becomes more uniform, with a larger Schottky contact area contributing dominantly to current conduction.

The much lower SBH values of the planar sample explain its limited operation range. The SiNW RF-Sputtering diode is less impacted by the effects of contact inhomogeneity throughout the operable range, with the lowest *m* and narrowest parameter distribution. As expected, the highest degree of inhomogeneity is exhibited by the SiNW E-Beam diode. As observed previously, its parameter distribution merges the behaviors of the planar and RF-sputtering samples. An important note is that, after a certain bias point, this sample’s current is given nearly exclusively by a single parallel diode, rendering its behavior nearly ideal. While this could be observed to some extent for the other samples, the bias interval is much larger for the SiNW E-Beam diode.

Since each individual nanowire essentially creates a Schottky contact with the deposited metal, the overall current flow has hundreds of thousands of components. Because an analysis of each sub-diode is unfeasible, we preferred employing our *p-diode* model [30] to give an overall qualitative assessment of the most important Schottky parameters. In particular, the *p_eff_* non-uniformity parameter offers an evaluation of the nanowires’ aggregation.

#### 3.2.2. Sensing Performances

**Figure 7:**
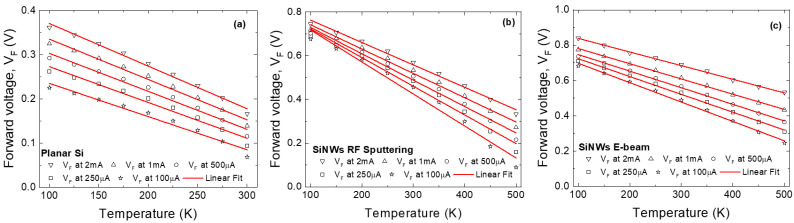
Forward voltage as a function of temperature for (**a**) Planar Si Schottky diode, (**b**) SiNWs Schottky diodes using RF sputtering as metal deposition, and (**c**) SiNWs Schottky diodes using E-beam as metal deposition, at several bias currents.

The *p-diode* analysis evinced that the samples are most suitable for temperature sensing at higher bias ranges, where the effects of contact inhomogeneity are least impactful. As such, the forward voltage—temperature dependence was determined, for each SBD, at various constant bias currents in the 0.1–2 mA range. Results, alongside associated linear fitting, are presented in Figure 7. The parameters of interest for the fitting process are the resulting slope (S) and coefficient of determination (R^2^), which yield sensitivity and degree of linearity, respectively (the higher, the better). Table 2, Table 3 and Table 4 give these parameters for each of the constant bias levels.

Because of its much lower SBH, the planar diode’s suitability for temperature sensing was only assessed in the 100–300K range. Even so, the sensitivity is low, with poor linearity, achieving a peak at a bias current of 100 µA.

For the SiNWs RF Sputtering sample, a considerable increase in sensitivity is achieved at 100 µA, while the most linear behavior was recorded for 2 mA. The increased SBH allowed evaluation of this sample’s performance in the 100–500K interval, yielding a substantially improved operable range, compared to its planar diode counterpart.

Following the *p-diode* modeling conclusions for the SiNWs E-beam sample, its suitability was also evaluated over the 100–500K range. The sensing performances of this diode are noticeably superior in terms of linearity. However, due to the overall lower SBH values and the larger distribution, sensitivity levels are closer to the planar sample.

Table 5 presents a comparison of our results with recent literature. To the best of our knowledge, there are no results reported on silicon Schottky diode-based temperature sensors, fabricated using silicon nanowires.

A clear superiority in terms of operable temperature range is evinced for the SiNW-based sensors, where the 100–500K interval is at least two times wider than planar counterparts. Linearity is comparable with state-of-the-art for the E-beam deposited sample, even though this parameter should degrade naturally as the temperature domain increases [9]. Lastly, the comparison evinced the need to explore alternative Schottky metals [4,25], as they yield different Schottky barrier values, which directly influence sensitivity.

Overall, the diodes with silicon nanowire contacts enable a much wider sensing domain than their conventional, planar counterpart, with better performances in terms of both sensitivity and linearity. While the characterization results tend to favor the RF Sputtering technique as superior, yielding more uniform devices, with much lower saturation currents, temperature sensing linearity was best by far for the E-beam sample.

## 4. Conclusions

This paper presented wide temperature range-capable silicon Schottky diodes for use as temperature sensors. Nanowires were obtained on the semiconductor’s epitaxial layer in order to increase the effective contact area and yield larger Schottky barrier heights. Two batches were fabricated, with Ti/SiNWs contacts. The metal was deposited either by RF sputtering or E-beam evaporation. SEM and XRD investigations were carried out, confirming proper nanowire layout and crystallinity. A diffuse reflectance analysis confirmed the quality of the metal film deposition and indicated that the sputtered devices exhibited better coverage.

The main Schottky diode parameters (barrier height, ideality factor, saturation current, series resistance, and non-uniformity parameter) were determined from forward characteristics and used to evaluate the bias and temperature ranges where the samples can operate as sensors. The SiNWs RF-Sputtering diode exhibited the highest overall SBH with good contact uniformity at elevated bias currents. This enabled the device to reach sensitivities up to 1.46 mV/K. Conversely, the SiNWs E-beam sample, while more inhomogeneous, had sensitivities comparable to the reference planar Schottky diode, but reached peak linearity, with R^2^ higher than 99.5% for all investigated constant bias currents. Both structures could properly operate as temperature sensors over 100–500K intervals, a much wider domain than is common for silicon devices. The *p-diode* model was crucial in enabling the determination of sensing performances, since the SiNW-based devices act essentially as hundreds of thousands of sub-diodes working in parallel, aggregated in groups.

The findings presented in this paper evince the prospects of nanowire technology for extending device operable range and obtaining better sensing performances.

## Figures and Tables

**Table 1:** Sample *p-diode* model parameter ranges.

Sample	*m*	Φ_Bn_ Range [V]	*p_eff_* Range
Planar	5	0.016–0.5	20.9–3.11
SiNW RF-Sputtering	4	0.5–0.78	17.16–6.17
SiNW E-Beam	5	0.014–0.76	22.43–9.25

**Table 2:** Sensing performances for Planar Si Schottky diode.

Current	R^2^	Sensitivity, S (mV/K)
2 mA	0.968	0.75
1 mA	0.97	0.81
500 µA	0.976	0.86
250 µA	0.982	0.913
100 µA	0.989	0.964

**Table 3:** Sensing performances for SiNWs RF Sputtering Schottky diode.

Current	R^2^	Sensitivity, S (mV/K)
2 mA	0.99	1.02
1 mA	0.988	1.11
500 µA	0.985	1.21
250 µA	0.98	1.31
100 µA	0.974	1.46

**Table 4:** Sensing performances for SiNWs E-beam Schottky diode.

Current	R^2^	Sensitivity, S (mV/K)
2 mA	0.9978	0.772
1 mA	0.998	0.863
500 µA	0.997	0.94
250 µA	0.996	1.01
100 µA	0.995	1.1

**Table 5:** Silicon Schottky diode sensing performances. Comparison with the state of the art.

Technology	R^2^	S [mV/K]	*T*–Range [K]	Ref
Planar Schottky diode	0.996–0.998	1.65	208–388	[4]
Planar Schottky diode	0.995	1.7	223–423	[25]
Planar Metal/Si-on-insulator Schottky diode	-	1.5 to 1.8	233–398	[31]
Planar Schottky diode	0.989	0.964	100–300	This work
SiNW RF-Sputtering	0.974	1.46	100–500	This work
SiNW E-Beam	0.995	1.1	100–500	This work

## Data Availability

The original contributions presented in this study are included in the article. Further inquiries can be directed to the corresponding author.

## References

[1] Di Benedetto L., Licciardo G.D., Rao S., Pangallo G., Della Corte F.G., Rubino A. (2018). V2O5/4H-SiC Schottky Diode Temperature Sensor: Experiments and Model. IEEE Trans. Electron Devices.

[2] Kim C.K., Lee J.H., Choi S.M., Noh I.H., Kim H.R., Cho N.I., Hong C., Jang G.E. (2001). Pd- and Pt-SiC Schottky Diodes for Detection of H2 and CH4 at High Temperature. Sens. Actuators B Chem..

[3] Mansoor M., Haneef I., Akhtar S., De Luca A., Udrea F. (2015). Silicon Diode Temperature Sensors-A Review of Applications. Sens. Actuators A Phys..

[4] Basov M. (2021). Schottky Diode Temperature Sensor for Pressure Sensor. Sens. Actuators A Phys..

[5] Wang Y., Tian J., Wang B., Wan L., Jin Z. (2023). The Design of a 1–6 GHz Broadband Double-Balanced Mixer. Micromachines.

[6] Dutta S., Sinha S., Panda A. (2015). Application of a Schottky Diode as a Temperature Sensor. J. Phys. Sci..

[7] Rugen S., Bödeker C., Kortazar I., Larrazabal I., Friedrichs P., Kaminski N. Investigation of the Turn-on Behaviour of Silicon Pin-Diodes and SiC-Schottky-Diodes and Its Impact on the Anti-Parallel IGBT. Proceedings of the 2016 18th European Conference on Power Electronics and Applications (EPE’16 ECCE Europe).

[8] Gambino J.P., Hsu L.L., Lee M.A., Seshan K., Junction H., Turene F.E. (1992). On-Chip Temperature Sensor Utilizing a Schottky Barrier Diode Structure. U.S. Patent.

[9] Pascu R., Pristavu G., Brezeanu G., Draghici F., Godignon P., Romanitan C., Serbanescu M., Tulbure A. (2021). 60–700 K CTAT and PTAT Temperature Sensors with 4H-SiC Schottky Diodes. Sensors.

[10] Rao S., Di Benedetto L., Pangallo G., Rubino A., Bellone S., Della Corte F.G. (2016). 85-440 K Temperature Sensor Based on a 4H-SiC Schottky Diode. IEEE Sens. J..

[11] Pascu R., Craciunoiu F., Pristavu G., Brezeanu G., Kusko M. (2017). Oxide Trap States versus Gas Sensing in SiC-MOS Capacitors–The Effect of N- and P- Based Post Oxidation Processes. Sens. Actuators B Chem..

[12] Qi Y., Lai K., Lv H., Qi B., Zhao Y. (2021). Investigation on a Novel SiC Schottky Barrier Diode Hydrogen Sensor with Trench-Insulator Structure. Mater. Res. Express.

[13] Di Benedetto L., Landi G., Licciardo G.D., Neitzert H.C., Bellone S. (2015). Photovoltaic Behavior of V2O5/4H-SiC Schottky Diodes for Cryogenic Applications. IEEE J. Electron Devices Soc..

[14] Liu S.X., Wang T., Chen Z.Z. (2017). High-Performance of Al Nanoparticle Enhanced 4H-SiC MSM Photodiodes for Deep Ultraviolet Detection. IEEE Electron Device Lett..

[15] Leonardi A.A., Faro M.J.L., Irrera A. (2021). Silicon Nanowires Synthesis by Metal-Assisted Chemical Etching: A Review. Nano Mater..

[16] Romanitan C., Varasteanu P., Mihalache I., Culita D., Somacescu S., Pascu R., Tanasa E., Eremia S.A.V., Boldeiu A., Simion M. (2018). High-Performance Solid State Supercapacitors Assembling Graphene Interconnected Networks in Porous Silicon Electrode by Electrochemical Methods Using 2,6-Dihydroxynaphthalen. Sci. Rep..

[17] Liu Z., Yuan R., Wang S., Liao W., Yan L., Hu R., Chen J., Yu L. (2025). Skin-Inspired Self-Aligned Silicon Nanowire Thermoreceptors for Rapid and Continuous Temperature Monitoring. Nano Lett..

[18] Wilson P., O’Connor T., Boland F., Budri A., Moore Z., Phelan N., Patton D. (2025). The Utility of Skin Surface Temperature Measurement in the Prediction of Diabetic Foot Ulceration. J. Tissue Viability.

[19] Rouis A., Hizem N., Hassen M., Kalboussi A. (2022). Electrical Properties of Silicon Nanowires Schottky Barriers Prepared by MACE at Different Etching Time. Silicon.

[20] Nath P., Sarkar D. (2024). Effect of Temperature on the Electrical and Optical Properties of Silver (Ag) Assisted Electrochemically Etched Silicon Nanowires (SINWs). Braz. J. Phys..

[21] Yang W.F., Lee S.J., Liang G.C., Eswar R., Sun Z.Q., Kwong D.L. (2008). Temperature Dependence of Carrier Transport of a Silicon Nanowire Schottky-Barrier Field-Effect Transistor. IEEE Trans. Nanotechnol..

[22] Cheung K.W., Yu J., Ho D. (2018). A Novel Surface Area to Volume Ratio Estimation Technique for Nanohemisphere Contacted Schottky Barrier Structures. AIP Adv..

[23] Hu R., Liang L., Zhang S., Liu Z., Wang J., Yu L. (2024). Comprehensive Understanding of Schottky Barrier Tunneling FET Built upon Ultrathin Silicon Nanowire for Monolithic 3D Integration. ACS Appl. Nano Mater..

[24] Meng J., Li Z. (2020). Schottky-Contacted Nanowire Sensors. Adv. Mater..

[25] Efeoǧlu H., Turut A., Gül M. (2023). An Experimental Study: Dependence of Schottky Diode Parameters on Schottky Contact Area Size. Opt. Mater..

[26] Pascu R., Pristavu G., Mihalache I., Popescu M., Simion M., Craciun G., Varasteanu P., Vulpe S., Brezeanu G. Silicon Nanowires-Based Schottky Diodes. Proceedings of the 2024 International Semiconductor Conference (CAS).

[27] Romanitan C., Kusko M., Popescu M., Varasteanu P., Radoi A., Pachiu C. (2019). Unravelling the Strain Relaxation Processes in Silicon Nanowire Arrays by X-Ray Diffraction. J. Appl. Cryst..

[28] Kaganer V.M. (2016). Elastic versus Plastic Strain Relaxation in Coalesced GaN Nanowires: An X-Ray Diffraction Study. Phys. Rev. Appl..

[29] Hasan M., Huq M.F., Mahmood Z.H. (2013). A Review on Electronic and Optical Properties of Silicon Nanowire and Its Different Growth Techniques. SpringerPlus.

[30] Brezeanu G., Pristavu G., Draghici F., Pascu R., Corte F.D., Rascuna S. (2020). Enhanced Non-Uniformity Modeling of 4H-SiC Schottky Diode Characteristics Over Wide High Temperature and Forward Bias Ranges. IEEE J. Electron Devices Soc..

[31] Wang K., Lv L., Ma Q., Zhang G., Shen S. (2025). Mechanically Tunable Schottky Diodes Based on Silicon Microstructure Arrays via Flexoelectricity. Appl. Phys. Lett..

